# Specialist adult ADHD clinics in East Anglia: service evaluation and audit of NICE guideline compliance^†^

**DOI:** 10.1192/pb.bp.113.043257

**Published:** 2015-06

**Authors:** Rakesh Kumar Magon, Beena Latheesh, Ulrich Müller

**Affiliations:** 1 Hertfordshire Partnership University NHS Foundation Trust; 2 Cambridgeshire and Peterborough NHS Foundation Trust; 3 University of Cambridge

## Abstract

**Aims and method** To measure compliance with National Institute for Health and Care Excellence (NICE) recommendations in two adult attention-deficit hyperactivity disorder (ADHD) clinics and to guide further service development. We audited the case notes of 150 patients referred to adult ADHD clinics in East Anglia in 2010-2011 against NICE standards using an adapted version of the ADHD audit support tool.

**Results** We found good compliance with NICE standards for diagnosis, assessment and pharmacological treatment of adult ADHD. There was a failure in smooth transitional arrangements from child and adolescent mental health to adult ADHD services. Comprehensive treatment programmes addressing psychological, behavioural, educational and occupational needs were not well developed. Deficiencies were observed in conducting recommended physical examinations. Substance use was prevalent in almost half of ADHD patients.

**Clinical implications** Greater attention is needed in delivering better transitional arrangements and comprehensive treatment programmes for adult ADHD. More structured training with emphasis on ADHD-specific psychological interventions, physical examination and treatment of complex cases, especially with comorbid substance misuse, should be offered to clinicians.

Attention-deficit hyperactivity disorder (ADHD) is the most common neurodevelopmental disorder. Symptoms of ADHD persist into adulthood and 10-20% of children with a diagnosis of ADHD still meet diagnostic criteria in adulthood.^1^ The National Institute for Health and Care Excellence (NICE) guideline makes clear recommendations for assessment and management of ADHD in adulthood.^2^ An increasing number of mental health trusts in the UK are implementing this guideline and offer services for adults with ADHD.^3^

The Adult ADHD Research Clinic in Cambridge (with a satellite clinic in Peterborough) is a joint venture between the Department of Psychiatry at the University of Cambridge and Cambridgeshire and Peterborough NHS Foundation Trust (CPFT); this tertiary referral centre provided a diagnostic and treatment advice service for East Anglia until it was temporarily suspended in August 2011. In January 2013, a new National Health Service (NHS)-funded adult ADHD service was started by CPFT. Adult ADHD clinics of the Hertfordshire Partnership NHS Foundation Trust (HPFT) are part of secondary mental health services for adults in Hertfordshire providing comprehensive assessment, diagnosis and treatment. Patients stabilised with drug therapy are referred back to their respective general practitioner (GP) through a shared care agreement.

## Method

The study was conducted to measure compliance of current practice in two adult ADHD centres with recommendations in the NICE guideline and to further inform service development.

We audited the case notes of 150 patients who were referred to our adult ADHD clinics in Cambridgeshire and Hertfordshire in 2010–2011 against NICE standards using an adapted version of the ADHD (adult) audit support tool.^4^ The percentage of patients was calculated for whom selected relevant NICE standards were met. As a part of the audit, data on patterns of substance use in the ADHD population were also collected. The project had formal approval from both participating trusts.

## Results

There were a total of 150 ADHD adult patients selected consecutively for this audit from the two trusts (CPFT *n* = 100, HPFT *n* = 50).

### Demographics

The study sample (Table 1) was predominantly male (77%), young (66% were 18–30 years old) and White (84%). Ethnicity was not recorded in the case notes of 64 adult ADHD patients. Less than a third of the sample (*n* = 46) was in regular employment, with 51 patients in the unemployed category; the rest were in education (*n* = 41). Employment status was unknown for 12 patients. Fifty-seven ADHD patients had a forensic history.

### Transitional arrangements

There was failure in transition to adult ADHD services in 34 out of 53 (64%) cases known to child and adolescent mental health services (CAMHS) with an established ADHD diagnosis (Table 2). The reason quoted for non-transition in some cases was receiving treatment from abroad (6 cases); one patient did not want to continue the drug treatment and one patient was having difficulty with the drug treatment. The reason for non-transition in the remaining 26 patients was not known.

### Diagnosis and treatment

Good compliance was observed in using (and documenting) diagnostic criteria (DSM-IV and/or ICD-10)^5,6^ across both trusts (89% at CPFT and 100% at HPFT) and assessing psychosocial impairment along with patients’ needs, physical health history and coexisting conditions.

Drug treatment was the first line of treatment recommended for 80% (80/100) of the patients at CPFT and 94% (47/50) at HPFT. In HPFT, methylphenidate was the first drug tried in 79% (37/47) of cases and there was 100% compliance in initiation and titration of the methylphenidate and monitoring of side-effects. Before starting the drug treatment, a full mental health and social assessment was carried out for all patients. However, a full physical assessment prior to drug treatment was performed only in 11% of cases (5/47); some physical examination was carried out in 72% (34/47). Risk assessment for substance misuse and drug diversion was performed in the majority of cases (HPFT 94%; CPFT 100%). Of the patients taking methylphenidate, 10% (4/47) received routine blood tests. Antipsychotic use was observed in 3% of patients (5/150); none of these patients carried a diagnosis of psychotic disorder; one patient suffered with a comorbid tic disorder and indication of antipsychotic use in four other patients was not documented in case notes.

A medical or family history of serious cardiac disease, a history of sudden death in young family members or abnormal findings on cardiac examination was reported in only 7 patients, but an electrocardiogram (ECG) recording was performed for 14 patients.

A comprehensive treatment programme including drug treatment and addressing patients’ psychological, behavioural, educational or occupational needs was recommended by CPFT for 95% of adults with ADHD. However, drug treatment formed a part of comprehensive treatment programme in only 25% (12/47) of adults receiving treatment in HPFT, where a diagnostic and treatment service is established.

### Psychological intervention

Group or individual cognitive–behavioural therapy (CBT) to address the person’s functional impairment was considered

**Table 1:** Study sample demographics

Age group, years	18–30		31–65

Female	21		13

Male	78		38

Ethnicity			
White British		72	
Other White and Black background		9	
Other mixed background		1	
Asian		2	
Other ethnic background		2	
Unknown		64	

Employment status			
Regular employment		46	
Unemployed		51	
Student		41	
Unknown category and other		12	

for 15% (15/100) of the service users attending CPFT and 10% (5/50) of service users in HPFT. Psychological treatment was considered in the context of persisting functional impairment or patients’ choice as an alternative to drug treatment.

### Alcohol and substance misuse

Almost half of the sample diagnosed with ADHD used illicit or licit substances (45%; 67/150). Alcohol (19%; 28/150) and nicotine (17%; 26/150) were the most commonly used drugs. Patients also used caffeine (13%; 19/150); cocaine (10%; 15/150); cannabis (7%; 11/150); amphetamine (4%; 6/150); ecstasy (3%; 5/150); hallucinogens (0.6%; 1/150) and heroin (0.6%; 1/150). All adults with ADHD and comorbid substance use received treatment by clinicians with expertise in both ADHD and substance misuse management (HPFT) or were referred to the drug and alcohol team with a recommendation for starting ADHD drug treatment after the substance misuse management (CPFT). Overall, 85% (57/67) of patients using substances were offered drug treatment for ADHD and 15% (10/67) were referred or signposted to the drugs and alcohol team for treatment of alcohol dependence (*n* = 9) and opiate dependence (*n* = 1).

### Person-centred care

Written information about the illness and on the treatment and care was given, along with information on the availability of NICE guidance to the majority of patients (148/150).

### Organisational service

Organisational support in terms of training programmes covering diagnosis and management was present at both trusts. Local shared care arrangement between primary and secondary care was present at HPFT only. Under the shared care arrangement, a range of responsibilities from drug prescription to physical health monitoring and annual reviews is transferred when the specialist and the GP agree that the patient’s condition is reasonably predictable and the treatment regime has been specified. If the GP is not confident about undertaking this role, then they are under no obligation to do so. In such an event, the total

**Table 2:** Audit standards based on the NICE guideline and trust compliance

	Compliance with standards %	
Standards	HPFT	CPFT
Smooth transition for young people with ADHD receiving treatment and care from CAMHS	**67**	24^*^

Diagnosis should meet the diagnostic criteria in DSM-IV or ICD-10	100	89

Diagnosis process should include an assessment of the person’s needs, coexisting conditions, social, family and educational or occupational circumstances and physical health	100	100

Drug treatment should be the first-line treatment	94	80

Drug treatment should be started only under the guidance of a psychiatrist, nurse prescriber specialising in ADHD, or other clinical prescriber with training in the diagnosis and management of ADHD	100	^*^

Before starting drug treatment for adults with ADHD:	100	100
• A full mental health and social assessment should be performed		
• ECG if there is medical or family history of serious cardiac disease, a history of sudden death in young family members or abnormal findings on cardiac examination	100 (overusedin7cases)	^*^
• Risk assessment for substance misuse and drug diversion should be performed	94	100
• All recommended physical examination	**11** (34 received some physical examination)	^*^

Drug treatment for adults with ADHD should always form part of a comprehensive treatment programme that includes psychological, behavioural and occupational needs	**25**	95 (standard recommended on assessment^*^)

Antipsychotics should not be used for the treatment of ADHD in adults	90	100

Methylphenidate should be the first drug tried in adults with ADHD	78	^*^
People taking methylphenidate should not have:		^*^
• Routine blood tests	90	
• ECG	100	
During the titration phase, symptoms and side-effects should be recorded at each dose change by the prescriber	100	^*^
Adherence to NICE guidelines on methylphenidate use	100	^*^

Group or individual CBT to address the person’s functional impairment should be considered	**10**	**15**

Drug treatment for adults with ADHD who also misuse substances should only be prescribed by an appropriately qualified healthcare professional with expertise in managing both ADHD and substance misuse	100	^*^

Specialist ADHD teams should jointly develop training programmes for the diagnosis and management of ADHD for mental health, social care, forensic and primary care providers and other professionals who have contact with people with ADHD	100	100

Are there local shared care arrangements in place between primary and secondary care?	100	^*^

ADHD, attention-deficit hyperactivity disorder; CAMHS, child and adolescent mental health services; CBT, cognitive–behavioural therapy; CPFT, Cambridgeshire and Peterborough NHS Foundation Trust; ECG, electrocardiogram; HPFT, Hertfordshire Partnership University NHS Foundation Trust; NICE, National Institute for Health and Care Excellence.

* Standards for recommended therapeutic interventions were not applicable due to the limited service model (assessment and treatment advice only) at the time of the audit.

Highlighted low compliances (in bold) are discussed in the paper in detail.

clinical responsibility for the patient remains with the specialist in secondary care.

## Discussion

Overall, this first audit of adult ADHD services in East Anglia established good compliance with NICE guidance for assessment and treatment. This confirms that the implementation of NICE guideline recommendations for adults with ADHD is feasible and should therefore be rolled out and evaluated on a broader scale.

There was a failure in smooth transitional arrangements from CAMHS to adult ADHD services. Different reasons were quoted for non-transition in some cases, but for most patients the reasons were not known. The 2010 TRACK study, which looked into the transition policies, procedures and outcomes in Greater London,^7^ found that neurodevelopmental disorders such as ADHD did not come under the eligibility criteria for transfer from CAMHS to adult mental health services. These young people are either retained at CAHMS or referred to GPs or voluntary organisations. For patients with a childhood diagnosis of ADHD there should be a clear transitional arrangement that takes into account the fact that adolescents are the most critical group of patients and that lack of treatment during the transitional period typically results in increased morbidity in adulthood.^8^ Clear communication and transitional pathways between specialist adult ADHD services, CAMHS, community paediatricians and GPs are essential to ensure continuity of care for individuals with ADHD from adolescence to adulthood. Currently, HPFT has a policy and CPFT is developing a transitional pathway, with the aim of improving transitional arrangements.

NICE has explicitly expressed the need for full mental health and social assessment and full history and physical examination prior to the drug treatment.^2^ In our study good compliance was observed in using (and documenting) diagnostic criteria (DSM-IV and/or ICD-10) and assessing psychosocial impairment. A full mental and social assessment prior to starting the ADHD drug was carried out, but deficiencies were observed in conducting (or delegating) recommended physical examination. Interestingly, there was an overuse of ECG investigations. Such discrepancy in practice can be overcome by structured training on adult ADHD with emphasis on physical examination and the use of ECG. This can be complimented by filing a physical examination checklist in the patient file and developing a local protocol for the indication of ECG use in adult ADHD patients with a history (or family history) of cardiovascular problems.

Drug treatment was the first line of treatment in the majority of cases. However, attention is needed in delivering more comprehensive treatment programmes addressing psychological, behavioural, educational and occupational needs. Adult ADHD patients are commonly referred to existing psychological services embedded in local community mental health teams or Improving Access to Psychological Therapies (IAPT) services, which have limited expertise in managing ADHD symptoms and associated impairments. The main issues that affect adults with ADHD include poor skills in prioritising and organising workloads in the workplace and home environment, occupational and educational underachievement, poor interpersonal and social skills and low self-esteem.^9^ Although good evidence of the effects of psychotherapy in adulthood is sparse,^10^ new research supports the use of CBT programmes in adults with ADHD.^11–13^ In the UK, the Young–Bramham Programme^11^ provides an integrated approach for understanding ADHD, adjusting modules to the diagnosis and developing skills to cope with symptoms and associated impairments. The programme offers techniques based on psychoeducation, motivational interviewing, cognitive remediation and CBT.^11^

Use of antipsychotics was seen in 3% (5/150) of patients referred for ADHD assessment, despite the fact that NICE has ruled out the use of antipsychotic drugs in treatment of core symptoms of ADHD.^2^ The finding highlights the need for more effort in educating clinicians about safety and effectiveness of antipsychotics in ADHD. More comprehensive treatment programmes that address psychological, behavioural, educational and occupational needs should be established and encouraged through the development of local ADHD support groups and in partnership with the voluntary sector. These include anger management, occupational therapy, ADHD life coaching, inter-agency liaison and working with voluntary sector providing employment support and counselling services.

Several longitudinal studies of children and adolescents with ADHD have demonstrated an increased risk of developing substance use disorder compared with matched controls.^14,15^ Factors such as novelty-seeking personality traits, increased impulsivity, self-medication for ADHD symptoms^16^ and comorbid disorders such as conduct disorder^14,17^ and bipolar disorder^18^ increase the risk of developing substance use disorder in this population. Adults with ADHD are more likely to be past or current users of substances and use these substances in greater amounts. They are also more likely to receive treatment for previous alcohol and drug use disorders.^19^

In our sample, substance use was prevalent in almost half (45%) of the ADHD patients. Patients with substance use disorder were appropriately referred to the addiction team and/or managed by clinicians with expertise in treating both ADHD and substance misuse as per the NICE guideline. It is important that mental health professionals receive appropriate training in assessment and management of ADHD with comorbid substance use disorder. Magon & Müller^20^ discuss treatment studies in this area and provide a treatment algorithm to guide clinicians in the management of adult ADHD comorbid with different forms and severities of substance use disorders.

The national Prescribing Observatory for Mental Health (POMH-UK) launched a new Quality Improvement Programme (QIP) in 2013 focusing on prescribing for ADHD in children, adolescents and adults (www.rcpsych.ac.uk/pomh). The baseline audit on prescribing for ADHD was concluded and results published in a report in September 2013; the report is not available externally, but more information can be obtained by contacting POMH at pomh-uk@rcpsych.ac.uk. The QIP project will generate UK-wide data on prescribing for adults with ADHD and help to identify gaps in service provision.
